# Monocyte-Derived Macrophages Contribute to Chitinase Dysregulation in Amyotrophic Lateral Sclerosis: A Pilot Study

**DOI:** 10.3389/fneur.2021.629332

**Published:** 2021-05-14

**Authors:** Nayana Gaur, Elena Huss, Tino Prell, Robert Steinbach, Joel Guerra, Akash Srivastava, Otto W. Witte, Julian Grosskreutz

**Affiliations:** ^1^Hans Berger Department of Neurology, Jena University Hospital, Jena, Germany; ^2^Jena Centre for Healthy Ageing, Jena University Hospital, Jena, Germany; ^3^Department of Anaesthesiology and Intensive Care Medicine, Jena University Hospital, Jena, Germany; ^4^Centre for Sepsis Control and Care, Jena University Hospital, Jena, Germany

**Keywords:** neuroinflammation, chitinases, macrophages, neurodegeneration, ageing

## Abstract

Neuroinflammation significantly contributes to Amyotrophic Lateral Sclerosis (ALS) pathology. In lieu of this, reports of elevated chitinase levels in ALS are interesting, as they are established surrogate markers of a chronic inflammatory response. While post-mortem studies have indicated glial expression, the cellular sources for these moieties remain to be fully understood. Therefore, the objective of this pilot study was to examine whether the peripheral immune system also contributes to chitinase dysregulation in ALS. The temporal expression of CHIT1, CHI3L1, and CHI3L2 in non-polarized monocyte-derived macrophages (MoMas) from ALS patients and healthy controls (HCs) was examined. We demonstrate that while CHIT1 and CHI3L1 display similar temporal expression dynamics in both groups, profound between-group differences were noted for these targets at later time-points i.e., when cells were fully differentiated. CHIT1 and CHI3L1 expression were significantly higher in MoMas from ALS patients at both the transcriptomic and protein level, with CHI3L1 levels also being influenced by age. Conversely, CHI3L2 expression was not influenced by disease state, culture duration, or age. Here, we demonstrate for the first time, that in ALS, circulating immune cells have an intrinsically augmented potential for chitinase production that may propagate chronic neuroinflammation, and how the ageing immune system itself contributes to neurodegeneration.

## Introduction

Amyotrophic Lateral Sclerosis (ALS) is a fatal and relentlessly progressive neurodegenerative disorder. Although, clinically characterized by the loss of both upper and lower motor neurons, it is a multi-systemic condition driven by several cell non-autonomous processes. Glial dysregulation in particular can exacerbate disease progression and is necessary for motor neuronal death to occur (1–3). Multiple lines of evidence have shown that this dysregulation extends to the peripheral innate immune system. Patient monocytes have a pro-inflammatory transcriptomic profile (4), secrete increased levels of pro-inflammatory cytokines (5), and can infiltrate the central nervous system (CNS) (6); furthermore, these alterations can influence disease progression. Crucially, monocytes can be readily sampled and differentiated to macrophages *ex vivo* and although ontogenetically different, monocyte-derived macrophages (MoMas) and microglia functionally complement each other (7). Studying macrophages may therefore help understand disease-associated inflammatory sequelae in the CNS.

In lieu of this, reports of elevated cerebrospinal fluid (CSF) levels of chitinases in multiple neurodegenerative conditions are particularly intriguing, as they are considered markers of chronic gliosis (8–10). The chitinases, including CHIT1, CHI3L1, and CHI3L2, belong to the family 18 glycosyl hydrolases and bind to chitin, a natural polysaccharide found in the coating of various pathogens with high affinity. Dysregulated chitinase levels have been noted in a range of non-infectious diseases, including asthma, chronic obstructive pulmonary disease, multiple sclerosis, and even Alzheimer's disease. Multiple lines of evidence suggest that chitinases aren't merely markers of disease status, but are active components of the immunological response in pathological conditions characterized by chronic inflammation.

In ALS, these moieties exacerbate neuroinflammation and directly affect neuronal viability (11–13). While studies using post-mortem motor cortex and spinal cord tissue from ALS patients have reported micro-and astroglial expression of CHIT1 and CHI3L1, respectively, the cellular origins of these targets remain to be fully understood. *In vitro* studies using healthy controls (HCs) have shown that the chitinases are produced by mature macrophages, wherein they display distinct temporal expression patterns (14–16). Therefore, the objective of this pilot study was to examine whether these cells also contribute to chitinase dysregulation in ALS. To do so, we examined the “baseline” expression of CHIT1, CHI3L1 and, CHI3L2 in non-polarized monocyte-derived macrophages (MoMas) in patients with ALS relative to HCs.

## Materials and Methods

### Participant Recruitment

All experimental procedures were approved by the local Ethics committee of the Jena University Hospital (Jena, Germany, Nr. 3633-11/12) and conducted in accordance with the Helsinki Declaration; written informed consent was obtained from all participants prior to enrollment. Patients with a diagnosis of either definite or probable ALS (as per the revised El-Escorial criteria) and HCs were consecutively recruited between January and July 2020 from the Departments of Neurology and Transfusion Medicine at the Jena University Hospital, respectively. Cerebrospinal fluid (CSF) was only available for ALS patients (7 of 8) as these patients underwent lumbar punctures (LPs) as part of their clinical examinations. Therefore, to enable between-group (healthy vs. disease) comparisons of CSF and plasma chitinase levels, we enlisted a second independent cohort of individuals termed non-neurological disease controls (NDCs) who were also undergoing lumbar punctures as part of their consultations at the Department of Neurology. Physical impairment was assessed using the Amyotrophic Lateral Sclerosis revised ALS Functional Rating Scale (ALSFRS-R) and calculated Progression Rate [PR; (48-current score)/disease duration in months]. The novel D50 progression model was used to ascertain disease aggressiveness and relative disease phase (17). Briefly, D50 is a summative descriptor for overall disease aggressiveness and refers to the time taken in months for a patient to lose 50% of functionality (ALSFRS-R score of 24 from a possible maximum of 48). It is calculated using iterative least-square fitting of available ALSFRS-R scores. Relative D50 (rD50) is an open-ended reference point that describes the individual disease course covered in reference to D50, wherein 0 signifies disease onset and 0.5 indicates halved functionality. Using rD50 allows the categorization of patients into contiguous disease phases: an early semi-stable Phase I (0 ≤ rD50 < 0.25), an early progressive Phase II (0.25 ≤ rD50 < 0.5), and late progressive and late stable Phases III/IV (rD50 ≥ 0.5).

Participants receiving immunomodulatory medication and/or suffering from an acute infection were excluded. All participants were also screened for HIV, Hepatitis B and C, and SARS-CoV-2 infection at the time of blood collection. Detailed genetic testing was not performed.

### Primary Human Monocyte Isolation, Culture, and Differentiation

Peripheral venous blood was collected from all participants in EDTA-K vacuum tubes (Sarstedt, Germany). Monocytes were isolated from 7.5 ml of freshly drawn blood *via* positive immunomagnetic selection using StraightFrom^®^ WholeBlood CD14 MicroBeads (Miltenyi Biotec, Germany) as per the manufacturer's instructions. Eluted monocytes were re-suspended in simple RPMI-1640 Glutamax medium, counted, and seeded at a density of 5 × 10^5^ cells/well of a 24-well plate and allowed to adhere for 2 h. Cells were gently washed with warm DPBS (Gibco) to remove unbound cells. From thereon, cells were cultured in “differentiation medium” supplemented with 20% v/v human serum (Sigma Aldrich), 1% v/v penicillin-streptomycin and 20 ng/ml of recombinant human M-CSF (BioLegend). Cells were cultured under standard conditions (5% CO_2_, 37°C) for 9 days with media changes performed every 2 days; cell lysates and supernatants were harvested for qRT-PCR and ELISA experiments, respectively, at days 1, 3, 6, and 9. Cell health and morphology were continuously tracked using brightfield microscopy.

### RNA Isolation and qRT-PCR

Cells were homogenized in QIAzol lysis reagent (Qiagen) at the specified time points and total RNA was isolated using the phenol/chloroform method. RNA quantitation and purity were spectrophotometrically assessed (ND-1000, Nanodrop, USA). RNA integrity was assessed on the QIAxcel Advanced System using the Qiaxcel RNA QC kit V2.0 (both QIAGEN). Samples with an RNA integrity number 6 ≥ were included for further analyses. An equal amount of RNA (200 ng) was reverse transcribed from each sample using the RevertAid First Strand cDNA Synthesis Kit in a final reaction volume of 20 μl. All qRT-PCR reactions were performed using the Brilliant III SYBR Green qPCR Master Mix (Agilent Technologies) on the Rotor-Gene 6,000 instrument (Corbett Research) with the following cycling parameters: 3 min of polymerase activation at 95°C followed by 40 amplification cycles (95°C for 10 s, 60°C for 60 s).

All primer pairs were designed to be exon-spanning and are detailed in Supplementary Table 2. Primer specificity was verified in preliminary experiments using melt-curve analysis and capillary electrophoresis to verify the presence of single PCR products at the correct size. The Pfaffl equation was used for relative quantification of gene expression; expression was calculated relative to the housekeeping genes *HPRT1* and *RSP18* and to HC samples at day 1.

### ELISA Analyses

Cell culture supernatants were harvested at the specified time points, centrifuged to eliminate cellular debris (400 × g for 10 min), and frozen at −20°C until further analyses. CSF and plasma were prepared by centrifugation (1000 × g, 15 min) within a maximum of 1 h from collection, aliquoted and stored at −80°C until use. All CSF samples were inspected for evidence of a traumatic puncture. CHIT1, CHI3L1, and CHI3L2 levels in were determined using commercially validated kits in accordance with the manufacturer's instructions. The kits used were as follows: CHIT1 and CHI3L2 from MBL Life Science and CHI3L1 from R&D Systems. All samples and standards were assayed in duplicate with intra- and inter-assay variation ≤10 and 15%, respectively. Absorbance was measured at 450/540 nm. Sample concentrations were extrapolated *via* 4 parameter logistic regression fitting of the standard curve.

### Statistical Analyses

Statistical analyses were performed using the SPSS (version 25.0) and GraphPad Prism software packages. The Shapiro-Wilk test was used to check for normal distribution. Correlations between continuous variables were assessed using the Spearman's test. Between-group comparisons were performed using either the Student's *t*-test or Mann-Whitney *U*-test. Mixed two-way ANOVAs were performed to assess the effect of group (ALS vs. HC) on chitinase expression over time. Assumptions for sphericity and homogeneity of variances and co-variances were met unless stated otherwise. The Greenhouse-Geisser adjustment was used to correct for violations of sphericity where necessary. All outliers were retained for analyses. Summary data are reported as the mean with either 95% confidence intervals (CI) or the standard deviation.

**For gene expression comparisons:** Analyses were performed on the log_2_-transformed fold-change ratios calculated using the Pfaffl equation.

**For secreted protein comparisons:** Analyses were performed on rank-transformed data (data were transformed for normalization). Studentized residuals for outliers noted prior to transformation were as follows: CHIT1 dataset 3.07 and 3.5 and CHI3L1 dataset 3.22. Two-tailed statistical significance was set at *p* < *0.05*.

## Results

The final cohort from which MoMas were generated included 8 patients with ALS and 8 HCs. ALS patients were significantly older than HCs (ALS = 60.5 ± 7.7 years vs. HC = 51 ± 7.9 years, *t* (14) = 2.403, *p* = 0.03) and both groups had a greater proportion of males (ALS = 6, HC = 7) than females. Four patients were receiving riluzole for ≥2 months at the time of sampling. None of the ALS patients had active cancer or manifest diabetes. The additional NDC cohort recruited to allow CSF chitinase analyses was representative of the HC cohort both in terms of age and sex distribution. Further demographic details are outlined in Table 1. Additional diagnostic information for the NDC cohort is provided in Supplementary Table 1.

**Table 1:** Participant demographics and clinical data.

	**ALS patients**	**Healthy Controls**	**NDCs**
*n*	8	8	7
Age (years) mean ± SD	60.5 ± 7.7	51 ± 7.9	53.8 ± 14.6
Males	6	7	5
Females	2	1	2
ALSFRS-R mean ± SD	38.1 ± 7.5	-	
PR mean ± SD	0.6 ± 0.4	-	
Disease duration (months) mean ± SD, range	18.1 ± 17.8, 7–60	-	
D50 mean ± SD	34.7 ± 19.6	-	
rD50 ± SD	0.25 ± 0.12	-	
rD50-derived Disease Phase I/II/III	4/4/-	-	
Bulbar onset	2	-	
Limb onset	6	-	

*ALSFRS-R, Amyotrophic Lateral Sclerosis Functional Rating Scale Revised; ALS, Amyotrophic Lateral Sclerosis; NDCs, non-neurological disease controls; PR, progression rate; rD50, relative D50*.

**Figure 1:**
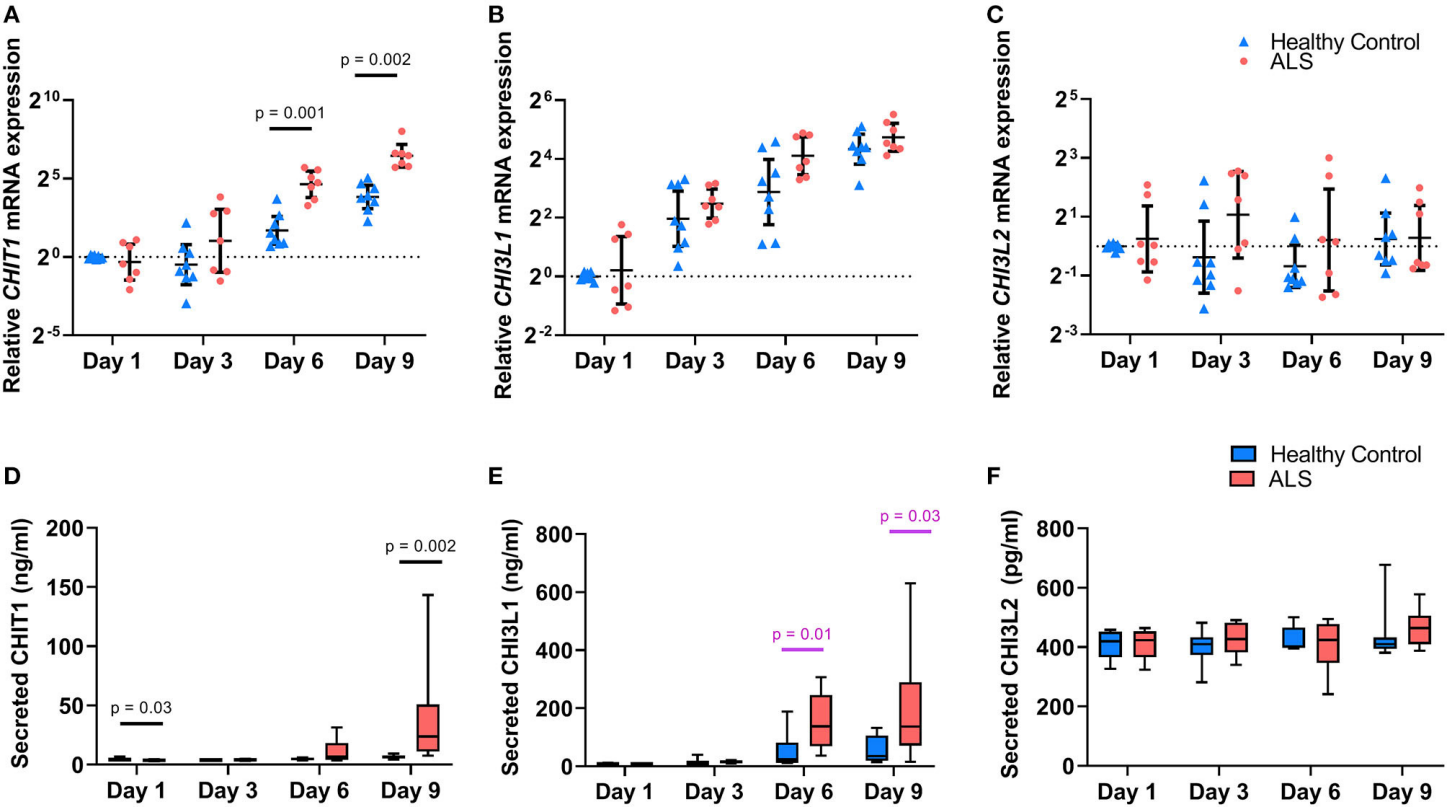
Chitinase expression in ALS and healthy control monocyte-derived macrophages. Relative expression of *CHIT1* **(A)**, *CHI3L1* **(B)**, and *CHI3L2* **(C)** in cell lysates from ALS patients (*n* = 8) and controls (*n* = 8) over time. Data are presented as individual scatterplots with the geometric mean and 95% confidence intervals. Dashed line at y = 1 corresponds to the relative expression of the calibrator samples (controls at Day 1). Protein levels of CHIT1 **(D)**, CHI3L1 **(E)**, and CHI3L2 **(F)** secreted by monocyte-derived macrophages from ALS patients (*n* = 8) and controls (*n* = 8) in culture over time. Data are presented as boxplots with whiskers indicating 95% confidence intervals. The effect of group and time on chitinase expression was assessed using a 2-way mixed ANOVA with significance set at *p* < 0.05. *P*-values are reported for statistically significant results; values reported in pink did not retain statistical significance after the inclusion of age as a covariate. Y axes for **(A–C)** are displayed in log_2_ scale.

As seen in Figure 1, CHIT1, CHI3L1, and CHI3L2 were detectable in MoMas from both ALS patients and HCs. However, they displayed distinct regulatory profiles. Temporal expression patterns were similar for CHIT1 (Figures 1A,D) and CHI3L1 (Figures 1B,E) in both ALS and HCs: relative gene and protein expression for both targets were minimal at earlier time-points, increased over time, and peaked on Day 9. However, no such temporal regulation was observed for CHI3L2 at either the transcriptomic (Figure 1C) or the protein level (Figure 1F) in either group. To illustrate, in HCs, mean secreted CHIT1 and CHI3L1 levels increased by 42.6 and 625%, respectively, from Day 1 to 9; conversely, for CHI3L2 only a 9% increase was observed. Indeed, secreted CHIT1 and CHI3L1 levels on D9 correlated significantly with each other but not with CHI3L2 (r_s_ = 0.69, *p* = 0.003).

Profound between-group differences were observed for CHIT1 and CHI3L1 at later time-points. Relative *CHIT1* expression was significantly higher in ALS MoMas on day 6 (*F*(2, 12) = 17.93, *p* = 0.001, partial η^2^ = 0.6) and day 9 (*F*(2, 12) = 15.42, *p* = 0.002, partial η^2^ = 0.56). This effect was recapitulated at the protein level, wherein a statistically significant *time* × *group* interaction was observed despite the inclusion of age as a covariate, thus underscoring the effect of group on CHIT1 levels over time (*F*(3, 39) = 4.97, *p* = 0.005, partial η^2^ = 0.27). ALS MoMas secreted significantly higher CHIT1 levels than HC MoMas on Day 9 (ALS = 39.4 ng/ml, [15.3, 53.5] vs. HC = 6.5 ng/ml, [−17.5, 30.6]) (*F*(1, 13) = 15.7, *p* = 0.002, partial η^2^ = 0.55), despite having lower levels on Day 1 (ALS = 3.6 ng/ml [3.1, 4.2] vs. HC = 4.6 ng/ml [4.1,5.2] (*F*(1, 13) = 5.77, *p* = 0.032, partial η^2^ = 0.31). An analogous trend was noted for CHI3LI: as seen in Figure 1B, relative *CHI3L1* expression was higher in the ALS group at all time-points and particularly so at Day 6. However, this effect did not reach statistical significance (*F*(1.6, 19.2) = 1.59, *p* = 0.23, partial η^2^ = 0.12). At the protein level however (Figure 1E), a statistically significant *time* × *group* interaction was noted (*F*(1.96, 27.43) = 7.09, *p* = 0.003, partial η^2^ =.34, Greenhouse-Geisser correction χ^2^(5) = 13.04, *p* = 0.02). Further, univariate analyses showed that ALS MoMas secreted significantly higher CHI3L1 levels than HC MoMas on Day 6 (ALS = 153.18 ng/ml, [91.6, 214.7] vs. HC = 51.2 ng/ml [−10.2, 112.7]) (*F*(1, 14) = 8.92, *p* = 0.01, partial η^2^ = 0.39), and Day 9 (ALS = 199.7 ng/ml [91.2, 308.2] vs. HC = 57.3 ng/ml [-51.2, 165.8]) (*F*(1, 14) = 5.83, *p* = 0.03, partial η^2^ = 0.29). Crucially, this group effect did not retain significance after the inclusion of age as a covariate (*F*(2.08, 26.99) = 2.60, *p* = 0.09, partial η^2^ = 0.17).

**Figure 2:**
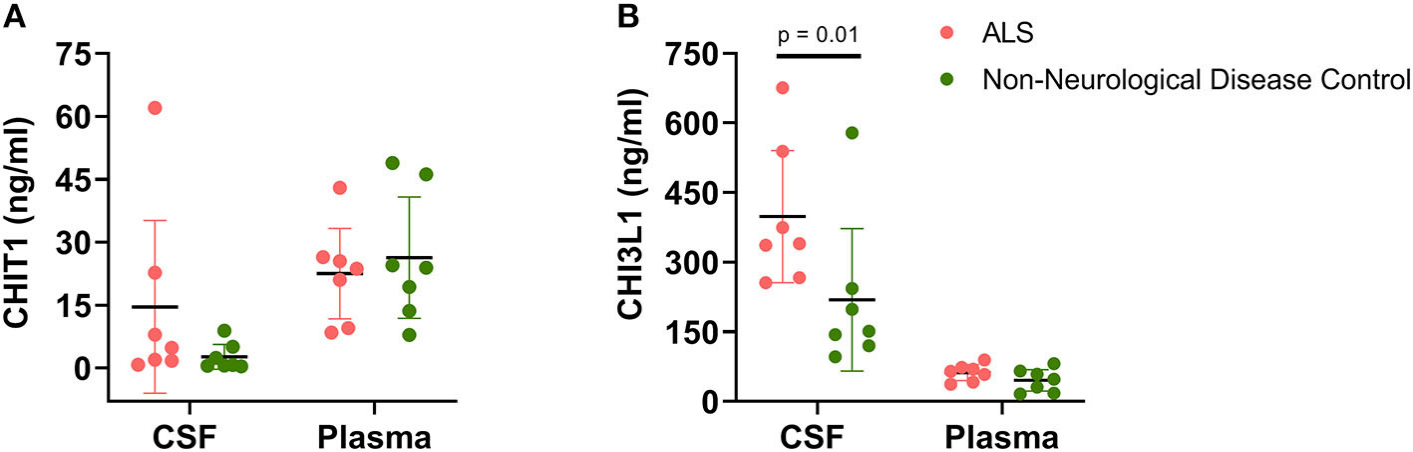
Chitinase levels in cerebrospinal fluid and plasma. Levels of **CHIT1 (A)** and **CHI3L1 (B)** were measured in the CSF and plasma of ALS patients (*n* = 7) and non-neurological disease controls (*n* = 7). Between-group comparisons were performed using the Mann-Whitney *U*-test with significance set at *p* < 0.05.

As seen in Figure 2, between-group biofluid analyses revealed that a disease-associated chitinase upregulation was only evident in CSF rather than plasma. Both CHIT1 and CHI3L1 plasma levels were largely similar between ALS patients and NDCs. Conversely, CSF CHI3L1 levels were significantly upregulated within the ALS group relative to the NDC group (ALS = 398.4 ng/ml, [256, 540.8] vs. NDC = 218.9 ng/ml [65.25, 372.5], *U* = 6, *p* = 0.017). While a considerable upregulation was also noted for CSF CHIT1 levels, the effect did not reach significance (ALS = 14.56 ng/ml, [−6.02, 35.14] vs. NDC = 2.65 ng/ml [−0.31, 5.62], *U* = 6, *p* = 0.017).

Finally, within the ALS group, no significant correlations were observed between secreted chitinase levels on Day 9 (this time-point was selected as this is when transcriptomic and protein expression peaked) and the total ALSFRS-R score, calculated PR, D50 and rD50 (*data not shown)*.

## Discussion

To our knowledge, this pilot study is the first to report a dysregulated chitinase profile in peripheral innate immune cells from ALS patients. By studying the transcriptomic and protein expression of key chitinases in non-polarized MoMas, we show here that macrophages in ALS have an intrinsically augmented capacity to secrete chitinases. To begin with, the temporal regulation patterns observed here are in keeping with previous studies; CHIT1 and CHI3L1 are minimally expressed in monocytes and highly upregulated during later stages of macrophage differentiation (14, 15). Conversely, CHI3L2 expression remains minimal across the differentiation process and is only upregulated as a result of stimulation (16). Here, the static and minimal CHI3L2 expression in both groups also serves to reinforce that the cells were at “baseline” and not stimulated as a result of the differentiation process itself. This, coupled with the absence of a group-associated effect, suggests that CNS rather than systemic immune cells likely contribute to the CHI3L2 elevations reported in the CSF of ALS patients. It is therefore unsurprising that the profound elevations we observed in CHIT1 and CHI3L1 expression in the ALS group were only evident at later time-points i.e. when cells were fully differentiated. Given the evidence that the chitinases are a feature of “M1-like” pro-inflammatory macrophages (18, 19), the upregulations observed here underscore how in ALS, peripheral myeloid cells are skewed toward a pro-inflammatory phenotype (4, 6). Indeed, monocytes from ALS patients are more readily differentiated toward an M1-like phenotype, wherein they produce higher levels of pro-inflammatory cytokines, including TNF-α and IL-6, than macrophages differentiated from HC monocytes (5). The data reported here are also interesting given that the chitinases themselves are active immune-modulators; for instance, stimulating monocytes with either CHI3L1 or CHIT1 resulted in the release of IL-8, MCP-1, and RANTES (20). Indeed, a “feed-forward” loop wherein the chitinases sustain neuroinflammation in ALS *via* their auto- and paracrine effects has already been postulated (12). For instance, Varghese et al. demonstrated that microglia appear to be the primary cellular source for CHIT1 in the CNS using murine cultures and that microglia themselves were susceptible to the effects of accumulated CHIT1, as they were chronically activated as a result of exposure (11). Another study also showed that conditioned medium from MoMas induced *CHI3L1* transcription and morphological changes in cultured human astrocytes (19). Crucially, chitinase exposure was shown to increase leukocytic migratory capacity across an *in vitro* blood-brain barrier (BBB) model (20). Therefore, one might hypothesize that neuronal death and aggregate deposition could trigger chitinase expression by glial cells, thus creating a chemotactic axis recruiting circulating monocytes. Finally, the monocytes, by virtue of their intrinsically augmented chitinase synthesis capacity, exacerbate the neuroinflammatory milieu upon differentiation. In keeping with this hypothesis, Steinacker et al. (8) reported that in post-mortem spinal cord tissue from ALS patients, CHIT1 immuno-staining was primarily observed in CD68+ve macrophages: no expression was noted in tissue from HCs.

The upregulations in CSF CHIT1 and CHI3L1 levels in ALS patients relative to NDCs are concordant with previous studies (8, 21). Indeed, CSF CHIT1 in particular is now considered a surrogate marker of microglial activity and recommended for the differential diagnosis of ALS (22). As also previously reported in the literature, we noted no significant between-group differences in plasma levels, which suggests that the chitinase dysregulation observed in ALS MoMas is more reflective of the inflammatory microenvironment in the CNS than the periphery. This is reinforced by our observation that monocytic expression of chitinases in both ALS and HCs was almost negligible.

Undoubtedly, the effect of age must be considered; the results observed here are to be expected, given that chitinase levels, particularly those of CHI3L1, increase with age and are potentially indicative of the wider “*inflammaging*” process (23). While further studies with age-matched cohorts are warranted, we posit that the effects of age and disease on chitinase expression are not mutually exclusive and should not be studied as such, as the contribution of “*immunosenescence*” to neurodegenerative conditions has been extensively reported (24).

The present study is not without its limitations, with the restricted sample size being foremost. While it sufficed for demonstrating proof-of-principle, these results warrant validation within a more sizeable cohort. We believe this also explains why no correlations were observed with clinical indices. Detailed information on existing chronic comorbidities like diabetes was only available for some individuals. However, these have also been reported to influence chitinase levels (25).

Next, the present study did not assess enzymatic CHIT1 activity as genetic information for *CHIT1* polymorphisms was not available for the cohort. The 24 bp duplication in exon 10 of the gene directly affects activity; heterozygous carriers display reduced activity and homozygous carriers display none at all (26). Therefore, the interpretation of these results would have been constrained, especially given that the prevalence of this polymorphism is almost 50% in European populations (27). However, given the observation that CHIT1 activity and protein levels are highly correlated, i.e., “elevated CHIT1 levels do not constitute inactive enzyme” (28), we posit that the results reported here are indeed evidence of a disease-associated CHIT1 upregulation in MoMas. Nevertheless, we recommend that future studies should include an assessment of CHIT1 activity.

Further studies with larger, age-matched and more representative cohorts can (1) help dissect the cumulative effect of age and disease on chitinase expression, (2) examine the implications for overall disease aggressiveness and acute activity, and (3) account for the dynamicity of the immune response by tracking chitinase expression across different disease phases.

## Data Availability Statement

The raw data supporting the conclusions of this article will be made available by the authors, without undue reservation.

## Ethics Statement

The studies involving human participants were reviewed and approved by Ethics committee of the Jena University Hospital (Jena, Germany, No. 3633-11/12). The patients/participants provided their written informed consent to participate in this study.

## Author Contributions

NG and JGr: study conceptualization and design. NG, EH, JGu, and AS: data acquisition, experimental execution, and troubleshooting. NG and TP: data analysis and visualization. NG: manuscript draft preparation. JGr, OW, RS and TP: revising the work for intellectual content. All authors contributed to the article and approved the submitted version.

## Conflict of Interest

The authors declare that the research was conducted in the absence of any commercial or financial relationships that could be construed as a potential conflict of interest.

## Abbreviations

ALS: Amyotrophic Lateral Sclerosis
ALSFRS-R: Amyotrophic Lateral Sclerosis Functional Rating Scale Revised
cDNA: complementary DNA
CHIT1: Chitotriosidase-1
CHI3L1: Chitinase 3-Like 1
CHI3L2: Chitinase 3-Like 2
CI: confidence interval
CNS: central nervous system
CSF: Cerebrospinal fluid
ELISA: Enzyme-linked immunoabsorbent assay
HCs: Healthy Controls
HPRT1: hypoxanthine phosphoribosyltransferase 1
IFNγ: Interferon gamma
LPs: lumbar punctures
MCP-1: Monocyte Chemoattractant Protein 1
MoMas: monocyte-derived macrophages
NDC: non-neurological disease controls
PR: Progression Rate
qRT-PCR: quantitative reverse transcription polymerase chain reaction
rD50: relative D50
RSP18: ribosomal protein S18
SD: standard deviation
TNF-α: Tumor Necrosis Factor alpha.
