# Supplementary material for: Monocyte-Derived Macrophages Contribute to Chitinase Dysregulation in Amyotrophic Lateral Sclerosis: A Pilot Study
Source: Front Neurol. 2021 May 14;12:629332. doi: 10.3389/fneur.2021.629332 (PMC8160083; doi:10.3389/fneur.2021.629332)
Supplement: Supplementary file 1 [file Table_1.docx]

**Supplementary Information**

**Supplementary Table 1: Diagnostic Information for independent Non-Neurological Disease Control Cohort**

| **Non-Neurological Disease Controls** | **n** |
| --- | --- |
| Idiopathic Intracranial Hypertension | 3 |
| Benign Fasciculations | 1 |
| Normal Pressure Hydrocephalus | 1 |
| Neurologically Healthy | 1 |
| Suspected pseudodementia | 1 |

**Supplementary Table 2: Primer Pair Details**

| **Target** | **Forward Primer** 5'-3' | **Reverse Primer** 5'-3' | **Product length** |
| --- | --- | --- | --- |
| *CHIT1* | CGCTTCACAACCCTGGTACA | AGCATCCACATAGGTCTGCC | 120 |
| *CHI3L1* | CCAGGAAAGCGTCAAAAGCAA | TTGATGGCATTGGTGAGAGGG | 141 |
| *CHI3L2* | GGAACCATAACTTTGATGGACTTG | GAGAAGCCTTTCCTTGGTGGA | 140 |
| *HPRT1* | GACCAGTCAACAGGGGACATA | GCTTGCGACCTTGACCATCT | 165 |
| *RSP18* | CCACGCCAGTACAAGATCCC | AAGTGACGCAGCCCTCTATG | 158 |
